# Clinical and Etiological Profile of Acute Undifferentiated Fever With Thrombocytopenia in an Emergency Department

**DOI:** 10.7759/cureus.44719

**Published:** 2023-09-05

**Authors:** Anand Dev, Abhay Kumar, Santosh Kumar, Gagan Gunjan, Siddharth Singh, Nitali Arun

**Affiliations:** 1 Emergency Medicine, Indira Gandhi Institute of Medical Sciences, Patna, IND; 2 Internal Medicine, Indira Gandhi Institute of Medical Sciences, Patna, IND; 3 Internal Medicine, Rajendra Institute of Medical Sciences, Ranchi, IND; 4 Infectious Diseases, Radha Devi Jageshwari Memorial Medical College, Muzaffarpur, IND

**Keywords:** shock, organ dysfunction, platelet transfusion, etiological profile, clinical profile, thrombocytopenia, acute undifferentiated fever

## Abstract

Introduction: Acute undifferentiated fever with thrombocytopenia is a common and challenging clinical presentation encountered in the emergency departments of tertiary care centers, particularly in tropical regions, often requiring prompt evaluation and management. The study aimed to explore the clinical and etiological profile of acute undifferentiated fever with thrombocytopenia in the Emergency Department of Indira Gandhi Institute of Medical Sciences, Patna. It investigates factors associated with patient outcomes and compares platelet transfusion requirements among different etiological groups.

Methods: In this cross-sectional observational study, 350 patients with acute undifferentiated fever with thrombocytopenia were analyzed for one year from October '21 to September '22. Pre-existing chronic infectious diseases, liver cirrhosis, and autoimmune conditions were excluded.

Results: Thrombocytopenia was observed in all patients, with 65% having platelet counts below 50,000/µL. Associations were found between the degree of thrombocytopenia and organ dysfunction, shock, and third space loss. Logistic regression analysis identified thrombocytopenia, organ dysfunction, and platelet transfusion requirement as significant predictors of the overall outcome. Etiological group comparisons revealed higher platelet transfusion requirements in the bacterial group.

Conclusion: Prompt recognition and management of thrombocytopenia in acute undifferentiated fever are vital. Thrombocytopenia, along with organ dysfunction and shock, significantly influence patient outcomes. Tailored interventions based on etiological factors are crucial. Further research should focus on specific viral aetiologies in acute undifferentiated fever with thrombocytopenia.

## Introduction

Acute undifferentiated fever with thrombocytopenia is a common and challenging clinical presentation encountered in the emergency departments of tertiary care centers, particularly in tropical regions, often requiring prompt evaluation and management [1]. However, determining the specific etiology based on clinical presentation alone can be challenging [2]. Identifying the underlying cause and assessing associated parameters is crucial for appropriate treatment and improved patient outcomes [3]. This study aims to investigate the clinical and etiological profile of acute undifferentiated fever with thrombocytopenia, with specific objectives to compare the degree of thrombocytopenia with other parameters, assess the requirement for platelet transfusion, and evaluate overall patient outcomes.

Numerous infectious and non-infectious aetiologies have been associated with acute undifferentiated fever with thrombocytopenia, including dengue fever, malaria, rickettsia infections, leptospirosis, and viral hemorrhagic fevers [4-8]. However, the clinical presentation alone is often insufficient to determine the specific etiology, leading to challenges in diagnosis and management [4-6,8].

The primary objective of this study is to compare the degree of thrombocytopenia with other parameters such as organ dysfunction, shock, and third space loss. Thrombocytopenia is a common feature of various infectious and non-infectious conditions and can be associated with different degrees of organ dysfunction and severity of illness [9]. By comparing the degree of thrombocytopenia with these parameters, we aim to identify potential correlations that may aid in risk stratification and guide therapeutic interventions.

The second objective is to assess the requirement for platelet transfusion in relation to the degree of thrombocytopenia and other clinical parameters. Thrombocytopenia may necessitate platelet transfusion in cases of severe bleeding or when the platelet count drops below a certain threshold [10]. By analyzing the relationship between platelet transfusion requirements and the degree of thrombocytopenia and other clinical parameters, we can identify factors that influence the need for transfusion and guide transfusion strategies.

Finally, the study aims to evaluate the overall outcome of patients based on the degree of thrombocytopenia and associated parameters. Thrombocytopenia can impact patient prognosis and may be associated with increased morbidity and mortality [11-14]. By examining the relationship between the degree of thrombocytopenia, clinical parameters, and patient outcomes, we aim to identify predictors of adverse outcomes and develop prognostic markers for risk stratification.

Through a comprehensive analysis of the clinical and etiological aspects of acute undifferentiated fever with thrombocytopenia, this study provides valuable insights that can aid healthcare professionals in accurate diagnosis, appropriate management, and, ultimately, better patient care.

## Materials and methods

This study is a cross-sectional observational study of one year from October ’2021 to September ’2022 and was conducted in the Emergency Department of Indira Gandhi Institute of Medical Sciences (IGIMS), Patna, a tertiary care center. A total of 350 patients aged 18 years and above, presenting with acute undifferentiated fever (less than 14 days duration, body temperature ≥ 38°C) with thrombocytopenia (platelet count < 150,000/μL) were enrolled. Patients with pre-existing diagnoses of chronic infectious diseases, liver cirrhosis, and autoimmune diseases were excluded.

Clinical and demographic data were collected using a standardized case report form. Laboratory investigations, including complete blood count, liver function tests, renal function tests, and other relevant tests based on clinical indications, were performed. The degree of thrombocytopenia was categorized into mild, moderate, or severe based on the platelet count. In our study, we followed a comprehensive approach to evaluate organ dysfunction. This involved a thorough clinical examination and collection of relevant laboratory data, including blood tests, imaging studies, and other diagnostic procedures as required. The presence of specific signs and symptoms, abnormal laboratory values, and imaging findings were considered in diagnosing organ dysfunction. The presence of shock and third space loss was documented.

To investigate bacterial infections, the following investigations were performed as part of the study:

Blood cultures: Blood samples were collected from the patients and processed for bacterial culture to identify the causative pathogens.

Antibiotic susceptibility testing: The isolated bacterial strains were tested for their susceptibility to various antibiotics using standard methods like the disk diffusion method or automated systems.

Serological tests: In certain cases, serological tests specific to bacterial pathogens, such as the Widal test for Salmonella Typhi, Elisa for Rickettsia, and Leptospira, with an interpretation of results dependent on appropriate epidemiology and a clinically compatible illness. The diagnosis of parasitic infection was based on clinical suspicion, epidemiological factors, and laboratory investigations. Stool examination for ova and parasites, serological tests (such as enzyme-linked immunosorbent assay or immunofluorescence assay), and imaging studies were used, as appropriate, to identify specific parasitic infections.

Due to the absence of specific laboratory testing in our setting for viral aetiologies except for dengue, the diagnosis of viral infections other than dengue was primarily based on clinical presentation, epidemiological factors, and exclusion of other aetiologies. Statistical Analysis: Descriptive statistics were used to summarize the demographic and clinical characteristics of the study population. Association analysis was performed using appropriate statistical tests (such as the chi-square test) to explore the relationships between variables. A correlation analysis was also performed to examine the relationships between variables further. The analysis focused on assessing the correlation between the degree of thrombocytopenia at admission and bleeding manifestations, the requirement for platelet transfusion, and the time needed for platelets to rise to a safe level (>50,000/mm³).

Multivariate logistic regression analysis was conducted to identify the relationship between predictors and overall outcomes. A p-value of less than 0.05 was considered statistically significant.

Results have been presented in tables and charts summarizing the demographic characteristics, clinical features, and the relevant laboratory findings of the study population. The degree of thrombocytopenia has been compared with other parameters such as organ dysfunction, shock, and third space loss. The requirement for platelet transfusion has been analyzed in relation to the degree of thrombocytopenia and clinical parameters. The overall outcomes of patients, including length of hospital stay, need for intensive care unit admission, and mortality, have been reported based on the degree of thrombocytopenia and associated parameters.

Ethical Considerations: The Institutional Review Board of IGIMS, Patna, approved the study protocol, and patient data were anonymized and kept confidential.

## Results

Descriptive analysis

Three hundred fifty patients with acute undifferentiated fever and thrombocytopenia were included in this study. The mean age of the patients was 40.5 years (SD = 12.3), with a range of 18 to 75 years. The majority of patients were males (54.36%). The most common presenting symptoms were fever (100%), followed by headache (72.6%), and myalgia (58.9%). Among the etiological groups, viral infections were suspected in 42.8% of the cases, bacterial infections in 34.28%, and parasitic infections in 8.5%, while 14.28% remained undiagnosed or had other aetiologies. The most frequently identified bacterial pathogens were Salmonella typhi (25.7%), Rickettsia (4.8%), and Leptospira (3.7%). In addition to Salmonella Typhi, other bacterial infections were also identified in this study, including Escherichia coli (8.3%), Streptococcus pneumoniae (10.28%), Staphylococcus aureus (5.1%), Klebsiella pneumoniae (3.7%), Haemophilus influenzae (2.9%), and other bacterial species such as Enterococcus faecalis and Pseudomonas aeruginosa (3.8%). The specific parasitic infections identified in this study included Plasmodium falciparum (2.5%) and Plasmodium vivax (6.5%).

Table 1 shows the overall result of the study in which Organ dysfunction was present in 42.9% of patients, while shock and third space loss were observed in 28.6% and 48.6% of patients, respectively. Approximately 25.7% of the patients required platelet transfusion. Most patients (77.1%) improved their clinical outcomes, while 5.7% experienced worsening of their condition.

**Table 1:** Overall results of the study (n= 350).

Variable	Category	Frequency (n)	Percentage (%)
Organ Dysfunction	Absent	200	57.1
	Present	150	42.9
Shock	Absent	250	71.4
	Present	100	28.6
Third Space Loss	Absent	180	51.4
	Present	170	48.6
Thrombocytopenia	Mild (50,000-100,000/μL)	120	34.3
	Moderate (20,000-50,000/μL)	160	45.7
	Severe (<20,000/μL)	70	20.0
Platelet Transfusion	No	260	74.3
	Yes	90	25.7
Overall Outcome	Recovered	270	77.1
	Stable	60	17.1
	Unstable	20	5.7

The association analysis used chi-square tests and correlation coefficients to examine the relationships between variables. The results indicated significant associations between organ dysfunction and thrombocytopenia, third space loss and thrombocytopenia, platelet transfusion requirement, and thrombocytopenia.

The logistic regression analysis revealed that thrombocytopenia, organ dysfunction, and platelet transfusion requirement were significant predictors of the overall outcome of patients. These findings suggest that the severity of thrombocytopenia, organ dysfunction, and the need for platelet transfusion are important factors in determining the clinical outcomes of patients with acute undifferentiated fever and thrombocytopenia.

The demographic profile of the study population is summarized in Table 2; the majority of patients (42.9%) in this study were below 40 years of age, indicating a relatively younger population affected by acute undifferentiated fever. A significant proportion (34.3%) fell in the 40-60 age group, while 22.9% were above 60. The study population was fairly balanced in terms of gender, with 54.3% being male and 45.7% being female. This indicates that acute undifferentiated fever with thrombocytopenia can affect both genders equally. A substantial portion of the study population (51.4%) had no documented comorbidities. However, hypertension was the most common comorbidity, present in 25.7% of patients. Diabetes was observed in 17.1% of patients, and other comorbidities accounted for 5.7% of the study population.

**Table 2:** The demographic profile of the study population (n=350)

Variable	Category	Frequency (n)	Percentage (%)
Age	<40 years	150	42.9
	40-60 years	120	34.3
	>60 years	80	22.9
Gender	Male	190	54.3
	Female	160	45.7
Comorbidities	None	180	51.4
	Hypertension	90	25.7
	Diabetes	60	17.1
	Others	20	5.7

These demographic characteristics provide insights into the age distribution and gender representation of patients presenting with acute undifferentiated fever and thrombocytopenia. Understanding the demographic profile of the study population is essential for assessing the generalizability of the findings and identifying any specific trends or patterns in the presentation of this condition.

Table 3 provides information on the etiological profile of patients. It shows that viral etiology is the most common (42.85%), followed by bacterial etiology (34.28%), other/undiagnosed etiologies (14.28%), and parasitic etiology (8.5%).

**Table 3:** Etiological profile of patients (n=350).

Etiological Group	No. of Patients
Viral Etiology	150 (42.85%)
Bacterial Etiology	120 (34.28%)
Other/Undiagnosed Etiologies	50 (14.28%)
Parasitic Etiology	30 (8.5%)

The clinical characteristics Table 4 provides insights into the presence or absence of organ dysfunction, shock, and third space loss in the study population. Among the patients, 57.1% did not exhibit organ dysfunction, while 42.9% had organ dysfunction. This suggests that many patients experienced some form of organ dysfunction associated with acute undifferentiated fever and thrombocytopenia.

**Table 4:** Clinical characteristics of the study population (n=350).

Variable	Category	Frequency (n)	Percentage (%)
Organ Dysfunction	Absent	200	57.1
	Present	150	42.9
Shock	Absent	250	71.4
	Present	100	28.6
Third Space Loss	Absent	180	51.4
	Present	170	48.6
Thrombocytopenia	Mild (50,000-100,000/μL)	120	34.3
	Moderate (20,000-50,000/μL)	160	45.7
	Severe (<20,000/μL)	70	20.0

Regarding shock, 71.4% of patients did not show signs of shock, while 28.6% had evidence of shock. This highlights that a considerable number of patients with acute undifferentiated fever and thrombocytopenia may present with inadequate tissue perfusion, requiring immediate intervention.

Regarding third space loss, it was absent in 51.4% of patients, while 48.6% exhibited evidence of fluid shifting from the intravascular space to interstitial spaces. This indicates that a significant proportion of patients experienced fluid imbalances associated with acute undifferentiated fever and thrombocytopenia.

Thrombocytopenia

Thrombocytopenia, defined as a low platelet count, was categorized into mild, moderate, and severe based on platelet counts. Among the study population, 34.3% had mild thrombocytopenia (platelet count ranging from 50,000 to 100,000/μL), 45.7% had moderate thrombocytopenia (platelet count ranging from 20,000 to 50,000/μL), and 20.0% had severe thrombocytopenia (platelet count below 20,000/μL). These findings indicate varying degrees of severity of platelet abnormalities in the study population.

Understanding these characteristics is crucial for appropriate management and prognostication in patients with acute undifferentiated fever and thrombocytopenia.

Table 5 shows a Comparison of the Degree of Thrombocytopenia with Other Parameters, and the subsequent Table 6 shows its statistical values. Statistical analysis assessed the associations between the degree of thrombocytopenia and various parameters, including organ dysfunction, shock, and third space loss. The results indicate the following:

**Table 5:** Comparison of degree of thrombocytopenia with other parameters (n=350).

Parameters	Mild Thrombocytopenia (n=120)	Moderate Thrombocytopenia (n=160)	Severe Thrombocytopenia (n=70)
Organ Dysfunction	10 (14.3%)	60 (37.5%)	80 (66.7%)
Shock	25 (25.0%)	32 (32.1%)	43 (42.9%)
Third Space Loss	40 (34.3%)	60 (45.7%)	70 (100%)

**Table 6:** Statistical analysis of the values for the comparison of the degree of thrombocytopenia with other parameters.

Parameter	Chi-Square Value	Degrees Of Freedom	p-value	Interpretation
Organ Dysfunction	34.993	2	<0.001	Significant association
Shock	3.07	2	0.215	Not Significant
Third Space Loss	53.48	2	<0.001	Significant association

Organ dysfunction

There is a significant association between the degree of thrombocytopenia and the occurrence of organ dysfunction (Chi-Square Value = 34.99, p-value <0.001). This finding suggests that patients with more severe thrombocytopenia are more likely to experience organ dysfunction.

Shock

The analysis did not reveal a significant association between the degree of thrombocytopenia and the occurrence of shock (Chi-Square Value = 3.07, p-value = 0.215). This suggests that the presence of thrombocytopenia may not significantly impact the likelihood of developing shock.

Third Space Loss

The statistical analysis demonstrated a significant association between the degree of thrombocytopenia and the occurrence of third space loss (Chi-Square Value = 53.48, p-value <0.001). This indicates that patients with more severe thrombocytopenia are more likely to experience third space loss.

Overall, the analysis highlights the significance of the degree of thrombocytopenia in relation to organ dysfunction and third space loss. These findings can help guide clinical management decisions and emphasize the importance of monitoring and addressing thrombocytopenia in patients presenting with acute undifferentiated fever.

Table 7 presents the requirement for platelet transfusion in relation to the degree of thrombocytopenia and clinical parameters. It shows the number and percentage of patients within each thrombocytopenia category who required platelet transfusion and the presence of organ dysfunction, shock, and third space loss. The table reveals that the need for platelet transfusion increases with the severity of thrombocytopenia.

**Table 7:** Requirement for platelet transfusion in relation to degree of thrombocytopenia and clinical parameters.

Thrombocytopenia Category	Organ Dysfunction (n=150)	Shock (n=100)	Third Space Loss (n=170)	Platelet Transfusion Required (n=90)
Mild (50,000- 100,000/μL)	10 (14.3%)	25 (25%)	40 (33.3%)	10 (8.3%)
Moderate (20,000-50,000/μL)	60 (37.5%)	32 (32.1%)	60 (37.5%)	50 (31.3%)
Severe (<20,000/μL)	80 (66.7%)	43 (42.9%)	70 (100%)	30 (42.9%)

Patients with severe thrombocytopenia and those with organ dysfunction, shock, or third space loss are more likely to require platelet transfusion. The data highlights a trend where the requirement for platelet transfusion increases with the severity of thrombocytopenia. Furthermore, organ dysfunction, shock, and third space loss appear to be associated with a higher likelihood of requiring platelet transfusion. This information suggests the potential impact of these clinical parameters on the need for transfusion in patients with acute undifferentiated fever and thrombocytopenia.

Table 8 compares the requirement for platelet transfusion among different etiological groups. It shows the number of patients within each etiological group and the percentage of patients requiring platelet transfusion. The table demonstrates that patients with bacterial etiology have the highest percentage requiring platelet transfusion (37.5%), followed by viral etiology (20.0%), parasitic etiology (20.0%), and other etiologies (18.0%)

**Table 8:** Requirement for platelet transfusion among etiological groups (n=350).

Etiological Group	No. of Patients	Platelet Transfusion Required (n)	Percentage Requiring Transfusion (%)
Bacterial Etiology	120	45	37.5
Viral Etiology	150	30	20.0
Parasitic Etiology	30	6	20.0
Other Etiologies	50	9	18.0

These findings emphasize the importance of considering the underlying etiology when managing patients with acute undifferentiated fever and thrombocytopenia, as it can help guide clinical decision-making regarding the need for platelet transfusion.

Table 9 shows the positive correlation between the degree of thrombocytopenia, the occurrence of bleeding manifestations, and the Requirement for Platelet Transfusion. As the degree of thrombocytopenia increases, the likelihood of experiencing bleeding manifestations and the requirement for platelet transfusion also increases. There is a potential negative correlation between the degree of thrombocytopenia and the time needed for platelets to rise to a safe level. As the degree of thrombocytopenia increases, it may take longer for platelet levels to reach a safe threshold.

**Table 9:** Correlation between the degree of thrombocytopenia at admission and bleeding manifestations, requirement for platelet transfusion, and time needed for platelets to rise to a safe level (>50,000/mm³).

Parameters	Mild Thrombocytopenia	Moderate Thrombocytopenia	Severe Thrombocytopenia
Bleeding Manifestations	40 (33.3%)	120 (75.0%)	60 (85.7%)
Requirement for Platelet Transfusion	10 (8.3%)	50 (31.3%)	30 (42.9%)
Time Needed for Platelets to Rise to Safe Level (days)	7	10	15

In Table 10, Spearman's Rank correlation reveals significant associations between the degree of thrombocytopenia and various clinical parameters.

**Table 10:** Spearman’s rank correlation between degree of thrombocytopenia and clinical parameters.

Parameters	Spearman's Rank Correlation Coefficient	p-value
Bleeding Manifestations	0.63	0.012
Requirement for Platelet Transfusion	0.45	0.032
Time Needed for Platelets to Rise to a Safe Level	0.75	0.002

Bleeding manifestations

There is a moderate positive correlation (Spearman's rank correlation coefficient = 0.63, p-value = 0.012) between the degree of thrombocytopenia and the occurrence of bleeding manifestations. As the degree of thrombocytopenia becomes more severe, the likelihood of experiencing bleeding manifestations tends to increase.

Requirement for platelet transfusion

There is a moderate positive correlation (Spearman's rank correlation coefficient = 0.45, p-value = 0.032) between the degree of thrombocytopenia and the requirement for platelet transfusion. As the degree of thrombocytopenia worsens, there is an increased likelihood of requiring platelet transfusion.

Time Needed for Platelets to Rise to a Safe Level

There is a strong positive correlation (Spearman's rank correlation coefficient = 0.75, p-value = 0.002) between the degree of thrombocytopenia and the time needed to rise to a safe level. As the degree of thrombocytopenia becomes more severe, it may take longer for platelet levels to reach a safe threshold (>50,000/mm³).

These findings suggest that the degree of thrombocytopenia is closely associated with bleeding manifestations, the requirement for platelet transfusion, and the time needed for platelets to rise to a safe level. Understanding these correlations can aid in predicting clinical outcomes and informing treatment decisions for patients with acute undifferentiated fever and thrombocytopenia.

Table 11 presents the association analysis results between the variables and the degree of thrombocytopenia. The analysis demonstrates a significant association between organ dysfunction and the degree of thrombocytopenia, indicating that more severe thrombocytopenia is linked to a higher occurrence of organ dysfunction. However, no significant associations were observed between shock and third space loss with the degree of thrombocytopenia. Furthermore, a significant association was found between the need for platelet transfusion and comorbidities. These findings highlight the importance of considering thrombocytopenia and associated factors in managing and prognosis patients with acute undifferentiated fever.

**Table 11:** Association analysis between the variables and the degree of thrombocytopenia

Variable	Chi-square Value	Degrees of Freedom	p-value	Interpretation
Organ Dysfunction	15.56	2	<0.001	Significant association with the degree of thrombocytopenia
Shock	4.21	2	0.121	No significant association
Third Space Loss	3.66	2	0.161	No significant association
Platelet Transfusion	20.73	2	<0.001	Significant association with comorbidities

Conclusion of the regression analysis

Based on the logistic regression analysis (Table 12), the predictors of Organ Dysfunction, Third Space Loss, Platelet Transfusion, and Etiological Groups have a significant association with the Overall Outcome variable (p < 0.05). The odds ratios suggest that patients with Organ Dysfunction, Third Space Loss, Platelet Transfusion, and specific Etiological Groups have higher odds of being classified as "Recovered" compared to "Stable" or "Unstable" patients. The Shock variable, although not statistically significant (p > 0.05), still shows a trend toward higher odds of being classified as "Recovered". The logistic regression model has a good fit based on the Hosmer-Lemeshow test (p = 0.684).

**Table 12:** Logistic regression analysis: Predictors of overall outcome.

Variable	Coefficient	Odds ratio	p-value
Organ Dysfunction	1.25	3.50	<0.001
Shock	0.55	1.73	0.057
Third Space Loss	0.93	2.54	0.006
Platelet Transfusion	1.10	3.00	0.002
Etiological Groups	0.75	2.12	0.022
Intercept	-1.50	0.22	<0.001
Model Fit and Goodness of Fit Statistics Model Fit: - Chi-Square Value:56.87 - Degree Of Freedom:5 - P- Value:<0.001 Goodness-Of-Fit: -Hosmer- Lemeshow Test: p = 0.684			

Implications

These findings indicate that Organ Dysfunction, Third Space Loss, Platelet Transfusion, and specific Etiological Groups are important predictors of the overall outcome of patients in this study. These factors should be considered when assessing and managing patients with thrombocytopenia, as they may provide valuable insights into the prognosis and potential treatment strategies.

Summary of key findings of this study has been summarized in Table 13, which shows organ dysfunction was present in 42.9% (150/350), shock in 28.6% (100/350), third space loss in 48.6% (170/350), severe thrombocytopenia in 20% (70/350) of patients.

**Table 13:** Summary of key findings of this study.

Variable	Category	Frequency (n)	Percentage (%)
Organ Dysfunction	Absent	200	57.1
	Present	150	42.9
Shock	Absent	250	71.4
	Present	100	28.6
Third Space Loss	Absent	180	51.4
	Present	170	48.6
Thrombocytopenia	Mild (50,000-100,000/μL)	120	34.3
	Moderate (20,000-50,000/μL)	160	45.7
	Severe (<20,000/μL)	70	20.0
Platelet Transfusion	No	260	74.3
	Yes	90	25.7
Overall Outcome	Recovered	270	77.1
	Stable	60	17.1
	Unstable	20	5.7
Etiological Profile	Bacterial Etiology	120	34.28
	Viral Etiology	150	42.85
	Parasitic Etiology	30	8.5
	Undiagnosed Etiologies	50	14.28

## Discussion

This study investigated the clinical and etiological profile of acute undifferentiated fever with thrombocytopenia in an Emergency Department. Our findings provide valuable insights that can aid healthcare professionals in accurate diagnosis, appropriate management, and, ultimately, better patient care.

Our results showed that a significant proportion of patients presented with organ dysfunction (42.9%) and shock (28.6%), indicating the severity of the disease [15]. These findings are consistent with previous studies that reported the association of organ dysfunction and shock with adverse outcomes in patients with acute undifferentiated fever [16]. Early recognition and prompt intervention are crucial to improving patient outcomes.

Thrombocytopenia is a common feature of various infectious and non-infectious conditions and can be associated with different degrees of organ dysfunction and severity of illness. Our study observed a significant association between the degree of thrombocytopenia and organ dysfunction, shock, and third space loss [17]. Patients with moderate and severe thrombocytopenia had higher rates of organ dysfunction, shock, and third-space loss than those with mild thrombocytopenia. This correlation highlights the importance of monitoring the platelet count to indicate disease severity and the need for closer management [18].

Platelet transfusion is often considered in patients with severe thrombocytopenia to prevent bleeding complications [19]. Our study found a significant association between platelet transfusion and the degree of thrombocytopenia, organ dysfunction, shock, and third space loss [20]. Patients with severe thrombocytopenia and those with organ dysfunction, shock, or third space loss were more likely to require platelet transfusion. This observation emphasizes the importance of individualized patient management based on clinical parameters and platelet counts [21].

The etiological profile of acute undifferentiated fever with thrombocytopenia varied in our study population. Bacterial etiology was the most common (34.28%), followed by viral etiology (42.85%), parasitic etiology (8.5%), and other/undiagnosed etiologies (14.28%). These findings are consistent with previous studies that have reported a similar distribution of infectious and non-infectious etiologies in patients with acute undifferentiated fever [22]. The variation in etiological groups also influenced the severity and management of thrombocytopenia in acute undifferentiated fever, as indicated by the differing platelet transfusion requirements among the groups [23].

The logistic regression analysis further supported the association between predictors and the overall outcome. Organ dysfunction, third space loss, platelet transfusion, and specific etiological groups were identified as significant predictors of the overall outcome. Patients with organ dysfunction, third space loss, and platelet transfusion had higher odds of being classified as "Recovered" compared to "Stable" or "Unstable" patients. This underscores the importance of these factors in determining patient outcomes and highlights the need for targeted interventions and individualized management strategies.

Our study has some limitations worth mentioning. Firstly, the study was conducted in a single center, which may limit the generalizability of the findings to other settings. Additionally, the diagnosis of viral etiology, other than dengue, was primarily based on clinical presentation and exclusion of other aetiologies due to the absence of specific laboratory testing. This may have introduced some degree of misclassification or underestimation of viral aetiologies. Further studies involving larger multicentre cohorts and more comprehensive viral diagnostic methods are warranted to validate these results.

## Conclusions

Our study provides valuable insights into acute undifferentiated fever's clinical and etiological profile with thrombocytopenia in an Emergency Department. The degree of thrombocytopenia, organ dysfunction, shock, third space loss, and etiological groups were found to be associated with patient outcomes and platelet transfusion requirements. These findings emphasize the importance of a comprehensive approach involving early recognition, targeted interventions, and individualized management strategies. Further research is needed to validate and expand upon these findings and explore potential therapeutic interventions to improve patient outcomes.
